# EMBEDDING REHABILITATION INTO CANCER CARE CONTINUUM: AN IMPLEMENTATION STUDY

**DOI:** 10.2340/jrm.v56.40855

**Published:** 2024-11-21

**Authors:** Fary KHAN, Bhasker AMATYA, Alaeldin ELMALIK, Krystal SONG, Demi DIAZ, Michael DICKINSON

**Affiliations:** 1 Department of Rehabilitation, Royal Melbourne Hospital and Peter MacCallum Cancer Centre, Parkville, Victoria; 2 Department of Medicine (Royal Melbourne Hospital), University of Melbourne, Parkville, Victoria; 3 Department of Clinical Haematology, Peter MacCallum Cancer Centre, and Royal Melbourne Hospital, Parkville, Victoria; 4 Patient Access & Flow, Peter MacCallum Cancer Centre, Parkville, Victoria, Australia

**Keywords:** cancer, rehabilitation, implementation, barriers, facilitators

## Abstract

**Objectives:**

To implement and evaluate a rehabilitation-inclusive service delivery model at a tertiary cancer hospital.

**Methods:**

The “Rehab-Toolkit”, a structured assessment tool comprising validated functional measures, was introduced in an inpatient cancer service. Consecutive inpatients were enrolled, and a Reach, Effectiveness, Adoption, Implementation, and Maintenance framework guided the analysis of barriers and facilitators for subacute care at clinic and system levels.

**Results:**

The implementation of the Rehab-Toolkit was incorporated into routine inpatient care. Major pre-implementation barriers included: absence of routine standardized functional assessment tools, limited coordination amongst acute and subacute care providers, low awareness of rehabilitation medicine amongst patients and professionals, and insufficient engagement of subacute care with interdisciplinary stakeholders in clinical decision-making. Following the intervention, there was a notable increase in awareness and the contributory role of subacute rehabilitation services, rehabilitation “needs” assessment, and referral pathways. Recommendations for process change included: development of clinical pathways, establishment of subacute referral systems and discharge coordinator roles, inclusion of subacute rehabilitation services in acute interdisciplinary team meetings, enhanced staff education and knowledge.

**Conclusion:**

Integration of rehabilitation services into cancer care can proactively manage functional morbidity. While the implementation process proved feasible and effective, robust process evaluation and longer term follow-up are necessary for sustained success.

## LAY ABSTRACT

A rehabilitation-focused care model using a structured assessment tool called the “Rehab-Toolkit” was introduced in the inpatient cancer service at a tertiary cancer hospital following analysis of institutional barriers and facilitators to subacute care. Initial challenges identified were: the lack of standard assessment tools for function, poor coordination between acute and subacute care providers, low awareness of the role of rehabilitation medicine, and limited involvement of subacute care in clinical decisions for complex discharge planning. Following the implementation of the toolkit, there was a significant increase in the awareness of input of rehabilitation services for complex survivorship issues, improved patient assessment, and usage of new referral pathways. Several process improvements were recommended, including: inclusion of clinical referrals and pathways in the electronic medical record, the new role of ward discharge coordinators, integration of rehabilitation physicians in interdisciplinary team meetings, and enhanced staff education. Overall, the incorporation of rehabilitation services into cancer care significantly enhanced patient functional health and overall management.

Cancer remains a global health challenge, characterized by complexity, high mortality rates, progressive nature, and uncertain prognosis (1). In 2020, an estimated 19.3 million new cases of cancer were diagnosed globally (2), with over 9.9 million cancer-related deaths (2). In Australia, the burden of cancer is significant, with 151,000 new cases and 49,000 cancer-related deaths recorded in 2021 (3). Lung and colorectal cancers are most prevalent, contributing to the overall cancer burden. There is a projected doubling of cancer incidence by 2040 (3). Treatments and supportive care requirements are resource-intensive, with substantial financial and productivity losses (6). Current therapeutic advances and improved cancer detection/diagnosis have enhanced the survival rates of patients. In Australia, the age-standardized 5-year relative survival of all cancers combined was 70.6% in 2015–2019, with further improvement projected in the coming years (3).

The clinical presentation of cancer varies depending on the type of cancer and associated symptoms. The World Health Organization (WHO) International Classification of Functioning, Disability and Health (ICF) (4) provides a framework for understanding the impact of cancer on individuals at different levels: *impairment* (body structure/function), *limitation in activity*, and *participation* within contextual factors (environmental and personal). Cancer-related impairments such as headache, seizure, neurocognitive dysfunction, muscle weakness, dysphasia, visual impairment, etc., can limit individual’s “activity” or function (decreased mobility, inability to self-care, etc.) and “participation” (work, family, social reintegration) (5). The ICF model offers a holistic approach to cancer care, encompassing the perspectives of both healthcare providers and cancer survivors. By addressing the complex interplay between impairments, activity limitations, and participation restriction, the ICF model facilitates comprehensive assessment and management of cancer-related symptoms and functional impairment.

Fig. 1 provides a simulated case example illustrating the application of the ICF model in the context of cancer care, emphasizing the importance of a multidimensional approach to address the diverse needs of cancer survivors.

**Fig. 1:**
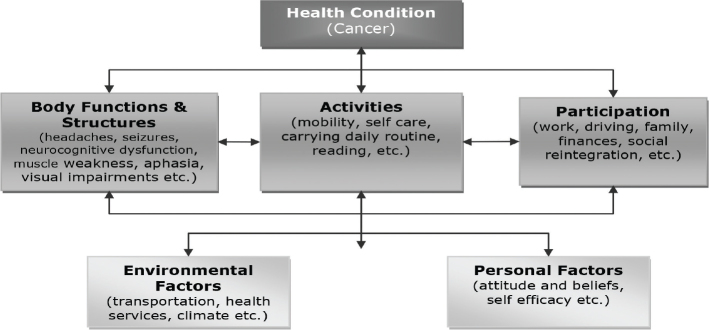
Interactions between the components of the ICF in cancer.

Cancer care is multifaceted, necessitating comprehensive coordinated care across hospital and community settings. However, existing care systems are often fragmented and hinder access to timely essential interventions and services when needed. Whilst cancer patients typically receive clinical assessments for the impact of oncological treatment on their disease, the assessment of their functional difficulties is often overlooked (6, 7), despite many cancer-related impairments being amenable to rehabilitation. Importantly, the risk of psychological distress in cancer patients is closely linked to their level of disability rather than the cancer diagnosis itself. Rehabilitation plays a crucial role in cancer management, with strong evidence for the positive impact of comprehensive rehabilitation programmes (8–12). Further, the provision of interdisciplinary prehabilitation enables patients to prepare, maximizes their resilience to treatment, and improves longer-term health (13). The failure to provide appropriate rehabilitation services to patients who could benefit can result in unnecessary physical and psychological suffering, and poorer QoL (10, 12).

Recognizing the significance of “function”, the WHO identifies this as the third crucial health indicator after mortality and morbidity, as many clinical decisions for an “optimal” therapeutic approach are based on the patient’s functional status (13, 14). Consequently, assessing functional status assumes critical importance in routine clinical practice, not only for optimizing therapeutic approaches but also for improving patient health outcomes and alleviating the healthcare burden. Further, early low-functional capacity is associated with poor treatment outcomes, including increased complications and mortality (7, 13, 14). Timely identification of functional difficulties identifies patients’ service needs, “need-based” level of care and interventions, timely referrals to required services (including rehabilitation), setting priorities and resource allocation, reduces hospital length of stay (LOS), and serves as a benchmark for monitoring longer term functional changes (7).

The initiation of a rehabilitation service at a tertiary Cancer facility identified gaps in the care continuum for many cancer patients. These included: lack of consistent standardized methods for assessing and reporting function and a systematic approach for documenting rehabilitation needs; delays in referrals to appropriate subacute services (subacute rehabilitation and/or aged care services); gaps within complex discharge planning and goal setting. This quality improvement initiative addressed these gaps by integrating pathways for patients across the hospital and community, tailored to their clinical needs through the implementation of a structured rehabilitation screening tool to identify deficits in patient function, barriers, and facilitators in service delivery, and evaluating the feasibility and effectiveness of the programme.

## METHODS

### Participants and setting

This study was conducted at the Peter McCallum Cancer Centre (PMCC), a tertiary referral centre that provides acute inpatient and ambulatory cancer care, including home services and palliative care with community partners. The project was approved by the institutional Human Research Ethics Committee (HREC/94235/PMCC).

### Procedure

A mixed-method process evaluation approach was applied, which included the following three phases: pre-implementation, implementation, and post-implementation.

*Pre-implementation phase:* A service resource-mapping exercise included a review of medical records of 22 consecutively admitted inpatients conducted prior to the project initiation. Information collected included rehabilitation assessment and functional status, goal-setting processes, patterns of referral to subacute services (Rehabilitation and/or Aged Care), complex discharge planning, need for services, and discharge destination.

The project team members (FK, AE, BA) collated information to identify barriers and facilitators in rehabilitation service provision through routine weekly interdisciplinary complex care meetings attended by acute cancer care clinicians (oncologists, surgeons, allied health (AH), and nurses). The rehabilitation physician joined these meetings during this phase. Information on barriers and facilitators identified through these meetings was recorded and transcribed verbatim by the principal investigators (FK, BA) for thematic analysis.

A Staff Information Sheet regarding the Rehab-Toolkit was then provided to all applicable staff. In addition, a series of ward/in-service education sessions involving information on the importance of functional assessment for medical, nursing, and AH staff were conducted. The Hospital Executive sponsored 2 x 1-day special “summit” sessions for relevant clinical staff across the hospital to discuss barriers to patient flow, including lack of referral processes for patients for subacute services, complex discharge planning, and community integration. All feedback was collated from these sessions and shared with participants.

*Implementation phase:* A series of information sessions by the rehabilitation physicians highlighted the role of rehabilitation and the Rehab-Toolkit see (Box 1) to medical, nursing, and AH staff during regular interdisciplinary meetings and forums. Further, didactic information sessions were conducted, using standardized presentations to demonstrate and describe the Rehab-Toolkit contents, assessment, and reporting processes. Sessions on what a rehabilitation service could offer patients were conducted by the rehabilitation physicians with Consumer Support and other services in the organization. In addition, rehabilitation information brochures were provided to patients, and the rehabilitation assessment tools and community support information were available on the wards and clinics. The Rehab-Toolkit documented information and was incorporated into the electronic medical record (EMR) system. The rehabilitation physicians commenced regular attendance at the weekly complex discharge and team planning meetings.

A total of 50 acute cancer patients were randomly recruited from the inpatient wards as part of ongoing Enhanced Recovery After Surgery (ERAS^®^) (between April and August 2023). One rehabilitation physician assessed the patients using Rehab-Toolkit on admission and discharge (see “Measures” section below) as part of routine clinical practice, with minimal burden to the patients. These assessments took approximately 20–30 min. Staff did not prompt patients, but assisted those who had difficulty with completing the questionnaires. Information concerning therapy, discharge planning, and other relevant clinical information was discussed with participants during these assessments. This was shared with the treating clinical team.

Further, an electronic referral pathway system with relevant patient criteria and suitability for subacute services was incorporated into the hospital EMR as a single point of entry for receipt of referrals across the organization and precinct. This included linkage with a Rehabilitation Medicine Consultative Liaison Service at the precinct (adjacent to the cancer hospital). A “hub and spoke” system of referrals for rehabilitation was charted and patients from the cancer facility were referred back to community (close to home wherever possible) across the state of Victoria, Australia. In addition, new roles for discharge coordinators in the cancer wards were commenced, supported by the rehabilitation physicians.

*Post-implementation phase:* A medical record review of randomly selected patients (*n* = 10) discharged from the various wards (performed within 12 weeks) analysed the post-implementation status of rehabilitation assessment, function, and service provision. Further, the primary researchers (FK, AE) discussed with staff the rehabilitation plans, including functional assessment and implementation of the Toolkit and its effectiveness at various forums. Staff feedback on barriers and facilitators for uptake were documented using qualitative techniques. A Department of Health survey was sent to all clinical staff to assess the outcomes of the ERAS programme (this included subacute services).

### Measures

The Rehab-Toolkit included a set of measures to capture various aspects of patient need, function, and well-being (Box 1).

Box 1. The Rehab-Toolkit

This comprised a suite of instruments to measure the patient’s function, participation, and health-related quality of life and includes:

- Clinical Functioning Information Tool (ClinFIT)
- Eastern Cooperative Oncology Group (ECOG) Performance Status scale
- European Quality of Life Scale (EQ-5D-5L)
- Functional Assessment of Cancer Therapy-General (FACT-G) (version 4)
- Clinical Frailty Scale (CFS)
- G8 Screening Questionnaire (G8-SQ)

*Clinical Functioning Information Tool (ClinFIT):* The WHO-endorsed universal non-proprietary measurement tool assessed patient function for rehabilitation needs (15). ClinFIT consists of 30 categories that define a minimum set of information on “functioning” and disability for collation across health conditions along the continuum of rehabilitation care: 9 items in the “Body Functions” domain and 21 items in the “Activities and Participation” domain. The clinicians assessed functioning problems patients experience as reflected in each ClinFIT item on an 11-point numerical rating scale, ranging from 0 = no problem to 10 = complete problem.

*Eastern Cooperative Oncology Group (ECOG) Performance Status scale:* assessed the patient performance status using a 6-point scale (0 = fully active to 5 = dead) (16).

*European Quality of Life Scale (EQ-5D-5L*) (17): assessed the overall QoL in 5 health dimensions: mobility, self-care, daily activity, pain/discomfort, and anxiety/depression. Items for these dimensions were rated across 5 ordinal levels (no problems to extreme problems). The sixth item within the scale assessed the participant’s current overall health using a visual analogue scale (VAS) from 0 (the worst health state they can imagine) to 100 (the best health state on that day they can imagine).

*Functional Assessment of Cancer Therapy-General (FACT-G) (Version 4) (**18**):* assessed the general QOL that reflects symptoms or problems associated with cancer patients across 4 subscale domains: Physical Well-Being, Social/Family Well-Being, Emotional Well-Being, and Functional Well-Being. The measure yields information concerning total QoL including physical, social, emotional and functional domains.

*Clinical Frailty Scale (CFS) (**19**):* a 9-point scale, graded the degree of fitness and frailty (1 = “very fit” to 9 = “terminally ill”) based on clinical judgement. It focuses on items that can be readily observed without specialist training (such as mobility, balance, use of walking aids, and the ability to eat, dress, shop, cook, do banking) (20).

*G8 Screening Questionnaire (G8-SQ) (**21**):* screened the frailty. A total patient score ranges from 0 (heavily impaired) to 17 (no impairment), with a cut-off score of ≤ 14 points suggesting a full geriatric evaluation requirement.

### Project evaluation

**Fig. 2:**
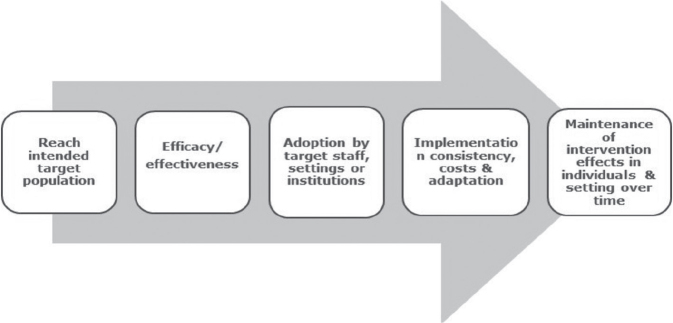
Five steps of programme evaluation using the Reach, Effectiveness, Adoption, Implementation, Maintenance (RE-AIM) framework. Source: Estabrooks (22), Song et al. (25).

The *Reach, Effectiveness, Adoption, Implementation, Maintenance* (RE-AIM) framework (22, 23) evaluated the implementation strategy and uptake of the recommended best practice Rehab-Toolkit. The RE-AIM framework addresses key areas of process evaluation including: sampling, recruitment, reach, acceptability and quality of the intervention, barriers and facilitators, and contextual influences. This framework also highlights the relative strengths and weaknesses of the programme by evaluating 5 key domains (Fig. 2). Key evaluation questions for which data was collected in this study are summarized in Table I.

**Table I:** Process evaluation questions related to Reach, Effectiveness, Adoption, Implementation, Maintenance (RE-AIM) domains and associated data collection tools

RE-AIM domains	Data collected	Key evaluation questions	Data collection tools
Reach	Number and proportion of staff participating in the Rehab-Toolkit implementation process	How is the target population reached? How well did the Rehab-Toolkit reach all those potentially eligible?	Description of data collated within the Rehab-Toolkit
Effectiveness	Intervention details & impact of Rehab-Toolkit on important outcomes (such as LOS, discharge destination)	Did the programme achieve its intended objectives? What intervention activities took place? Who conducted intervention activities?	Service and resource mapping Rehab-Toolkit information session, clinical process changes Descriptive data in study design Evaluate outcomes (LOS, number of referrals, referral outcomes)
Adoption	Number and proportion of staff who are willing to initiate a programme Adherence & attitudes of staff to Rehab-Toolkit	Was Rehab-Toolkit adopted by the treating clinical staff?	Qualitative barriers & facilitators from interdisciplinary group discussions Medical record review Quantitative outcome measures (Rehab-Toolkit) Plan strategies to enable and sustain change
Implementation	Staff fidelity to various elements of Rehab-Toolkit (consistency of delivery, time, adaptations made during delivery)	To what extent was the Rehab-Toolkit implemented as planned? Was the programme relevant (i.e., goal-directed & useful)? What were the barriers & enablers to programme delivery? Were there adaptations made during programme delivery? What were the areas of the programme that need improvement? What inputs/resources were allocated for programme implementation? How did external factors influence programme delivery? Was the structure or logic of the programme appropriate?	Number of information sessions to different disciplines & interdisciplinary forum Audit of Rehab-Toolkit & medical clinical progress notes Qualitative barriers & enablers feedback from interdisciplinary team discussions Clinical process changes Hospital inpatient rehabilitation programme & staff resources Discussion of issues with staff key managers including medical, nursing, & AH staff in existing interdisciplinary meetings
Maintenance	Extent to which Rehab-Toolkit becomes part of routine organizational practices & policies	Is long-term implementation feasible? Were the data used to change practice? What were the long-term benefits for participants?	Ongoing discussion of patient flow issues in existing interdisciplinary forums with key managers including medical, nursing, & AH staff Evaluate Rehab-Toolkit in future longer-term studies

AH: allied health [Adapted from: Song et al 2018 (25)].

### Statistical considerations

Descriptive summary statistics were generated for patient demographic and clinical characteristics. Qualitative analyses of staff barriers and facilitators from the staff group discussions were analysed, coded, and interpreted using thematic analyses. Thematic analysis was based on an inductive process that allows for themes to emerge and enables the management of large amounts of qualitative data in a credible and robust manner. Transcripts were individually read, “open” coded, and emergent thematic features were collectively discussed, categorized, and summarized under each topic domain by 2 investigators (FK, BA) until agreement was reached. Any discrepancy was resolved by group discussion amongst all investigators. As data were not normally distributed for all evaluated outcome measures, a series of non-parametric tests (Wilcoxon signed-rank test) was used to determine the differences between admission (T0) and discharge (T1). Effect size statistics (*r*) were calculated by dividing the z score by the square root of N (total number of cases) and assessed against Cohen’s criteria (0.2 = small, 0.5 = medium, 0.8 = large effect) (24).

## RESULTS

### Pre-implementation phase

The primary objective of the rehabilitation service was to improve patient care through: enhanced comprehensive functional assessment, delivery of evidence-based therapeutic interventions, strengthening patient education and support regarding their condition, and improving the care continuum outcomes. Prior to implementation, an evaluation of services and resources found deficiencies in functional assessment and referral procedures, predominantly relying on an ad-hoc, impairment-focused referral approach. The absence of standardized functional assessment criteria resulted in delays in access to rehabilitation interventions and discharge from the acute hospital. Inadequate communication between acute medical teams and rehabilitation professionals (such as AH, nurses, and rehabilitation physicians) led to variable documentation, ineffective goal setting, and delays in community reintegration. Despite the availability of AH in both hospital and community settings, there was a lack of an organized referral system for subacute services, with largely unplanned referrals and no formalized system to follow through with referrals to transition cancer patients to external services. Communication between treating clinicians and rehabilitation service providers was reactive and primarily focused on individual cases. Additionally, there was no mechanism in place for long-term follow-up post-discharge from the acute inpatient cancer setting. Project members (FK, AE, BA) convened interdisciplinary meetings, including those addressing complex patient discharges, to identify barriers and facilitators for effective and timely patient care and discharge. Thematic analyses of the information collated from these meetings highlighted various challenges in delivering rehabilitation services, as summarized in Box 2.

Box 2. Pre-implementation service mapping

- Rehabilitation, allied health, and patient support services operated in “silos”
- Rehabilitation physicians not included in goal-setting or care planning meetings in cancer wards
- Absence of comprehensive rehabilitation assessment process for medical management of disability and no rehabilitation clinics available to address survivorship issues
- Lack of integration of rehabilitation services within the acute care setting and between acute care and community care sectors
- No ward discharge coordinator role, resulting in poor communication between acute and relevant care providers
- No standardized tool to assess patient function and/or rehabilitation needs
- Lack of standardization of referral, triage, or rehabilitation care documentation
- Limited system for longer-term follow-up of patients in the community after discharge (other than acute cancer medication and surgery)
- Inadequate coordinated discharge services and communication with community partners
- Limited access to allied health services such as neuropsychology, speech and language therapy, etc.
- Ad hoc provision of information to patients in clinics according to clinician-assessed needs
- Limited access to patient self-management support services
- Marked variation in reported patient needs assessment (specifically function)
- Clinic time constraints; limited administrative and multidisciplinary clinical support
- Limited information and educational material regarding rehabilitation provided to patients

### Implementation phase

During this phase, qualitative data collection involved organized focus-group discussions within various meetings and forums to identify barriers and facilitators in service provision. A diverse array of healthcare professionals, including general and specialist clinicians, nurses, AH professionals, and hospital staff, participated in these discussions. Recurring themes that emerged from these discussions were systematically coded through iterative analysis and consensus agreement. Inductive analysis of these themes revealed 25 distinct barriers and 17 facilitators to service provision and care delivery, from a rehabilitation perspective. A primary systemic barrier identified was the fragmented care among multidisciplinary groups within the hospital, worsened by insufficient systems for subacute referral, triage, and discharge coordination, as well as limited communication platforms. Additionally, deficiencies in standardized patient assessment processes and data collection tools/systems were noted, leading to inconsistencies in information sharing and duplication of communication among care providers. Notably, significant overlap was observed among the barriers identified (Table II). Key facilitators and service components included: support of the hospital executive team and the patient flow coordinator for change management, the introduction of ward discharge coordinators to oversee interdisciplinary team communication, regular weekly interdisciplinary meetings with medical, nursing, and AH staff, formalizing community referral processes and follow-up, liaison with general practitioners and community partners, patient education and self-management support through goal-setting plans. Detailed insights into key barriers and facilitators are provided in Table II.

**Table II:** Key potential barriers and facilitators in rehabilitation service provision

Potential challenges/barriers	Potential facilitators/enablers
Unclear role/responsibilities of rehabilitation professionals within patient flow processes, expected timeframes/KPIs for discharge planning & liaison with external services Limited communication documentation for interdisciplinary input in the care processes for discharge planning Limited pre-admission discharge planning for planned surgical cases for rehabilitation Inadequate complex care planning and care coordination for sub-acute services Limited liaison of subacute services with interdisciplinary teams Limited rehabilitation resources, and coordinated timely referrals to subacute services & follow-up mechanisms Limited prehabilitation and pre-treatment workups prior to admission for some patients Inadequate patient and family communication concerning the discharge plan Limited skilled expertise in rehabilitation & geriatric services Poor integration of AH and rehabilitation physicians within acute cancer care team plans Lack of presence of a designated rehabilitation ward resulting in delay in referral No defined sub-acute referral pathways Acute care consultant-driven decision-making Limited documentation/assessment of patient rehabilitation needs Unplanned admissions from the community Limited robust process to ensure early identification of patient functional complexity & discharge barriers Delayed flagging of patient needs/issues (clinical, functional, social), e.g., NDIS Limited clinical pathways and protocols for rehabilitation treatments Lack of standardized patient assessment screening tool for function	Establishment of an Access and Flow Ward Clinical Group Discharge Coordinator service, introduction of a Discharge Coordinator and Rehabilitation Physician role Implementation of standardized screening and assessment tools to stratify patients’ discharge risk and ensure early and appropriate referrals Suitable discharge pathways available for patients including at home services Support and education to staff regarding rehabilitation needs and assessment Developed communication strategies to involve patients and families in decision-making Use of electronic platform for improved communication amongst team members and improved visibility of patient care across clinical groups Liaison with key stakeholders for improved access to rehabilitation services Refinement of the role and responsibilities of treating staff Daily meetings with discharge coordinators and treating teams Earlier engagement of ambulatory and sub-acute services Building a stronger relationship between cancer services and the subacute care team, including community rehabilitation Increase awareness of in-reach and rehabilitation consultation services for acute cancer patients Rehabilitation medicine involvement in the peri-operative context (functional assessment); peri-operative period to identify risks, suggest risk mitigation strategies, and prepare eventual disposition options Building stronger relationships with external inpatient subacute services Interdisciplinary stakeholder group meetings for resources, infrastructure, workflow, etc. Enhanced support of consumer forums Commencement of a new Rehabilitation Medicine Clinic to support AH staff

AH: allied health; NDIS: National Disability Insurance Scheme.

### Clinical outcomes

Table III presents descriptive statistics regarding patient demographics and disease characteristics for a randomly selected sample of 50 cancer patients in the implementation phase. The majority of participants were male (*n* = 28, 56%), and Caucasian (*n* = 44, 88%), with a mean age of 59.5 ± 14.8 years (range = 25.4–93.6). Colorectal cancer was the most common diagnosis among patients (*n* = 36, 72%), with an average disease duration of 1.7 ± 2.4 years. Approximately 68% of participants (*n* = 34) reported 1 or more comorbidities, with hypertension (*n* = 18, 36%) and diabetes (*n* = 9, 18%) being the most prevalent. The average length of stay was 15.9 ± 12.7 days.

**Table III:** Sociodemographic characteristics of participants (*n* = 50)

Characteristics	
Age, years, mean±SD (range)	59.5±14.8 (25.4-93.6)
Male, *n* (%)	28 (56.0%)
Ethnicity, Caucasian, *n* (%)	44 (88.0%)
Non-English-speaking background, *n* (%)	2 (4.0%)
Married/partner, *n* (%)	38 (76%)
Living, *n* (%)	
Alone	11 (22.0%)
Partner/family	39 (78%)
Education, *n* (%)	
Secondary	15 (30.0%)
Tertiary	34 (68.0%)
Employed, *n* (%)	
Full-time/part-time	21 (42%)
Unemployed/retired	27 (54%)
Transfer – independent, *n* (%)	16 (32%)
Mobility – independent, *n* (%)	15 (30%)
Personal activity of daily living – independent, *n* (%)	14 (28%)
Driving, *n* (%)	43 (86%)
Carer, *n* (%)	
Nil	41 (82%)
Informal services	3 (6%)
Formal services	6 (12%)
Length of hospital stay, days, mean±SD (range)	17.8±10.7 (5–62)
Discharge destination, *n* (%)	
Home/primary residence	31 (62%)
Institution (hospital, nursing home, etc.)	2 (4%)
Diagnosis, *n* (%)	
Gastrointestinal cancer	36 (72%)
Other cancers	14 (28%)
Time since diagnosis, years, mean±SD	1.5±2.4
Comorbidities, *n* (%)	
Hypertension	18 (36%)
Diabetes	9 (18%)
Impairments/symptoms, *n* (%)	
Cognitive impairment	2 (4%)
Dietary issues	28 (56%)
Speech	2 (4%)
Falls risk	1 (2%)
DVT risk	3 (6%)
Pain	48 (96%)
Fatigue	42 (84%)
Bladder issues	24 (48%)
Bowel issues	15 (30%)
Inpatient rehabilitation, *n* (%)	18 (36%)
Rehab referral, *n* (%)	20 (40%)

DVT: deep vein thrombosis; *n*: total number; SD: standard deviation.

More than two-thirds of patients required some form of supervision with their transfers (*n* = 34, 64%) and mobility (*n* = 35, 70%). The most common impairments reported included pain (96%) and fatigue (84%), followed by dietary issues (56%), bladder (48%) and bowel dysfunction (30%). Eighteen patients (36%) received inpatient multidisciplinary rehabilitation, and on discharge, 20 patients (40%) were referred to community-based rehabilitation programmes (see Table III).

### Change scores from admission to discharge

**Fig. 3:**
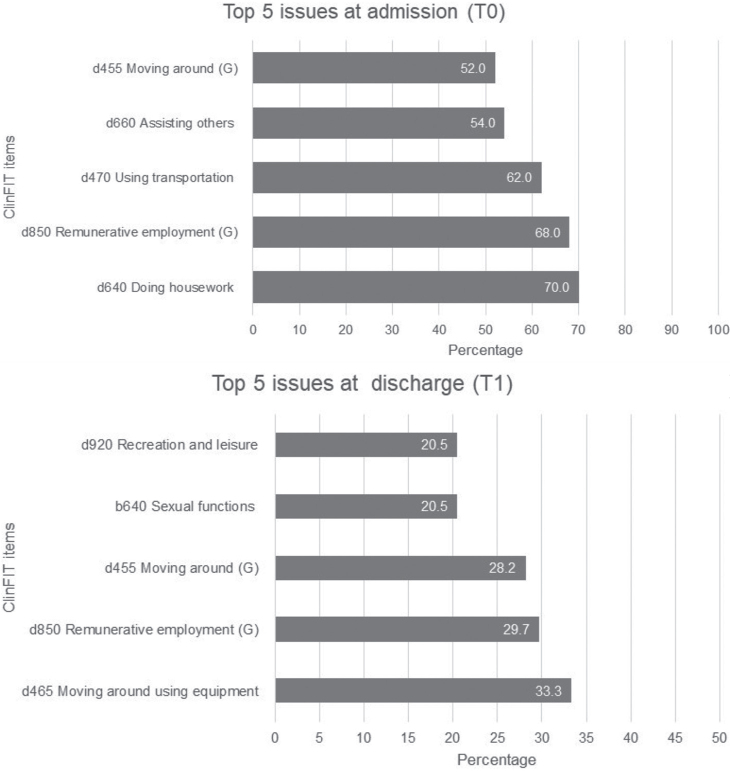
Top 5 most commonly affected Clinical Functioning Information Tool (ClinFIT) categories on admission (T0) and discharge (T1).

Fig. 3 illustrates the patient-reported problems attributed to their health conditions using ClinFIT within the domains of “Body Functions (B)” and “Activities and Participation (D)”. Most participants reported experiencing at least 1 issue related to the ClinFIT categories. The most prevalent ClinFIT category on admission (T0) was “d640 Doing housework” (70%), followed by “d850 Remunerative employment” (68%), and “d470 Using transportation” (62%). Conversely, on discharge, the most commonly reported issues were “d465 Moving around using equipment” (53.5%), followed by “d850 Remunerative employment” (29.7%), and “d455 Moving around” (28.2%) (Fig. 3).

**Fig. 4:**
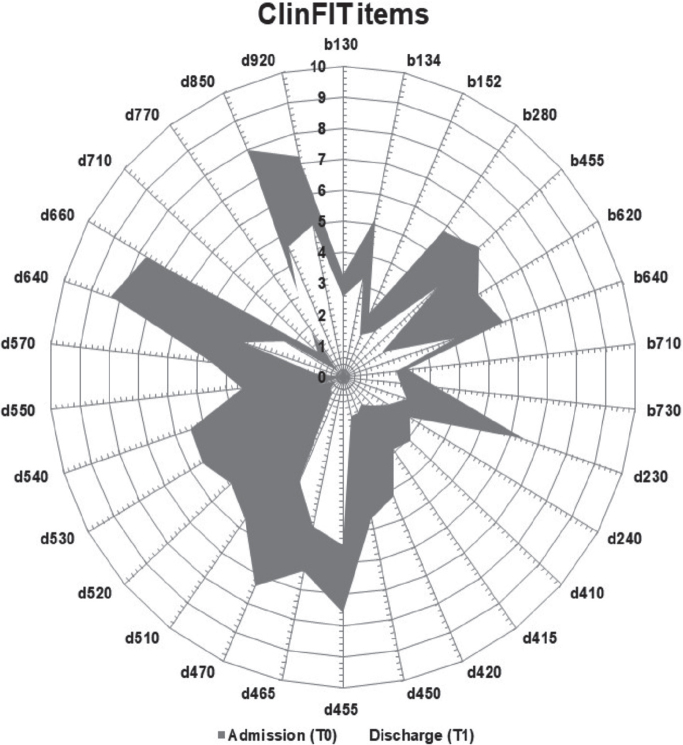
Composite radar chart illustrating the median scores for each ClinFIT item on admission (T0), and on discharge (T1)*. *The composite radar chart provides a graphic representation of the functional profile from the ClinFIT data. The 30-scale items are arranged as spokes of a wheel (codes outside the circumference), with ICF qualifiers from 0 = no problem to 10 = complete problem, running from the centre outwards. Dark-shaded area represents the median admission (T0) scores, and light-shaded area represents the median discharge (T1) scores. b130–b730 represent 9 ClinFIT “Body function” categories and d230–d920 represents 21 ClinFIT “activity and participation” categories.

Summary data for all outcome measures at different assessment points are presented in Table IV. Irrespective of cancer type and severity, the majority of participants demonstrated functional improvement from admission to discharge. There was a significant enhancement in participant discharge scores compared with admission ClinFIT scores (median [interquartile range]: 158 [102, 195] vs 72 [28, 93]; z value = –5.17, *p* < 0.001), with a medium effect size (*r* = 0.59). Most participants exhibited improvement across all items related to their body function (b) and activity and participation (d) domains (Fig. 4). Similarly, there were noteworthy improvements in participants’ QoL and overall health on discharge, as evidenced by significant EQ-5D-5L and overall health scores (*p* < 0.001 for all), with small to medium effect sizes (*r* = 0.28 to 0.54), and FACT-G Total scores (*p* < 0.001, *r* = 0.45) (see Table II). There were no changes observed in frailty (G8-SQ and CFS scores), although overall performance showed significant improvement (*p* = 0.002, *r* = 0.35) (Table IV).

**Table IV:** Change scores in subscales for measurement scale overtime

Scales	Admission (T0) *n* = 50 Median (IQR)	Discharge (T1) *n* = 39 Median (IQR)	z-values^	p-value*	Effect size (*r*)^#^
**ClinFIT** (total raw score) (0–300)	158 (102.5, 195)	72 (28, 93)	–5.17	**<0.001**	0.59
**ECOG** total (0–5)	0.0 (0, 1)	1.0 (0, 1)	–3.13	**0.002**	0.35
**G8SQ** (0–17)	14.0 (10.5, 15)	13.8 (11, 15)	–0.84	0.401	0.10
**CFS** (1–9)	3.0 (2, 4)	3 (3, 4)	–0.98	0.325	0.11
**FACT-G** total (0–108)	71 (81.8, 77)	81 (71, 87)	–3.96	**<0.001**	0.45
Physical well-being (0–28)	14 (8.6, 20.3)	25 (18, 26)	–4.83	**<0.001**	0.55
Social well-being (0–28)	24 (24, 26)	25 (18, 26)	–1.83	0.068	0.21
Emotional well-being (0–24)	15 (13, 16)	14 (12, 15)	–2.34	**0.019**	0.26
Functional well-being (0–28)	19 (12.6, 22)	20 (15, 24)	–2.03	**0.042**	0.23
**EQ-5D-5L**					
Mobility (1–5)	2 (1, 3)	0 (0, 1)	–4.44	**<0.001**	0.50
Self-care (1–5)	3 (1, 4)	1 (0, 2)	–4.75	**<0.001**	0.54
Daily activity (1–5)	3 (2, 4)	1 (1, 2)	–4.74	**<0.001**	0.54
Pain/discomfort (1–5)	2 (1, 4)	1 (0, 2)	–4.02	**<0.001**	0.46
Anxiety/depression (1–5)	1 (0, 2)	0 (0, 1)	–2.51	**0.012**	0.28
Overall health (0–100)	50 (30, 71)	75 (65, 85)	–3.89	**<0.001**	0.44

* Correlation significant at the <0.05 level (2-tailed) are shown in bold.

^ Wilcoxon signed-ranks test.

# Effect size statistics (*r*) Cohen’s criteria: 0.2 = small, 0.5 = medium, 0.8 = large effect.

CFS: Clinical Frailty Scale; ClinFIT: Clinical Functioning Information Tool; ECOG: Eastern Cooperative Oncology Group (ECOG) Performance Status scale; EQ-5D: Euro-Quality of life scale; FACT-G: Functional Assessment of Cancer Therapy-General; G8SQ: G8 Screening Questionnaire; IQR: interquartile range; *n*: total number of participants.

Similarly, on discharge, there was a significant improvement in the participants’ quality of life and overall health (EQ-5D-5L and overall health score): *p* < 0.001 for all with small to medium effect sizes (*r*=0.28–0.54) and FACT-G Total (*p* < 0.001, *r* = 0.45) (Table II). There was no change in their frailty (G8-SQ and CFS scores), but overall performance improved significantly (*p* = 0.002, *r* = 0.35) (Table IV). Overall, 18 participants received individualized interdisciplinary rehabilitation based on clinical need. The most commonly included rehabilitation intervention component was PT (*n* = 12) followed by a dietitian (*n* = 12). Most participants were discharged home (*n* = 36, 72%), 2 were discharged to other hospitals, and 1 was discharged to residential care. Fifteen patients were referred to the CBR programmes.

### Post-implementation phase

A medical record review of randomly selected patients (*n* = 10, discharged within 12 weeks) demonstrated improved consistency in assessment and clinical care processes, in particular, functional assessment (100%) to guide rehabilitation referrals. Further, discussions with various nursing, AH, and medical staff provided insights into their experiences with the rehabilitation process. Qualitative analysis of the group findings revealed persistent barriers to implementation, including constraints related to time and resources, access to the ClinFIT measure on the EMR, and persistent limited communication among healthcare providers. Some measures in the Rehab-Toolkit were not considered helpful or responsive (such as G8, CFS scores) in identifying specific domains of functional deficits. Despite these challenges, staff members expressed optimism about the “value-add” of subacute rehabilitation services and were motivated to address identified barriers. The provision of the Rehab-Toolkit facilitated improved patient assessment and goal setting, and more “tailored” rehabilitation interventions. Staff highlighted the role of the discharge coordinator and rehabilitation physician in improved streamlining of patient-flow processes, discharge planning, referrals, and team communication. Further, the commencement of a new Rehabilitation Medicine Clinic to support AH staff was deemed valuable.

### Project evaluation

The RE-AIM framework was used to evaluate the Rehab-Toolkit initiative. The project members (FK, AE, BA) explained the purpose of the rehabilitation initiative at various team meetings and reviewed the operational process of the project. The project team monitored the progress and discussed the status of their project with the relevant stakeholders. Further, the governance and leadership needed for the sustainability of the programme were discussed with the Executive and clinical leadership. The particular elements considered and used for analysis are summarized in Table V, and the impact of the quality improvement initiative is discussed below.

**Table V:** Evaluation of Rehab-Toolkit implementation initiative using Reach, Effectiveness, Adoption, Implementation, Maintenance (RE-AIM) framework

RE-AIM domains	Key factors	Pre-implementation	Implementation	Post-implementation	Impact of programme
Reach	Target population to be included in the initiative	Target population included all patients admitted to inpatient wards as a part of ongoing Enhanced Recovery After Surgery (ERAS^®^) programme Target population also included staff (medical, nursing, and AH)	Rehabilitation staff incorporated Rehab-Toolkit into routine clinical care and participated in implementing clinical change process	Assessed patients for rehabilitation service provision (e.g., referral, goal setting, discharge planning etc.)	Good reach of interventions to eligible populations
Effectiveness	Impact of intervention on important outcomes	Barriers and facilitators to subacute service provision identified	Patient assessments on admission and discharge using Rehab-Toolkit Staff education Patient information	Increase in functional assessment for rehabilitation needs of patients; triaging, service provision, & improved team communication	Good impact on the provision of rehabilitation interventions and referrals. Longer-term studies required to evaluate patient outcomes post-discharge, including QoL
Adoption	Adoption of intervention by staff and services	Exploration of staff barriers and facilitators to rehabilitation service provision using interdisciplinary group meetings	Initiation of structured patient assessments and information delivery to patients by staff, provision of referral systems, triaging and improved team communication, complex discharge planning	Rehab input well received by patients, with no unwanted effects or staff burden Ward discharge coordinators and patient flow services supported rehab team and were motivated to overcome barriers	Overall positive attitude of acute cancer care clinicians towards initiative suggests good adoption, consistent with post-implementation results of the study
Implementation	Extent to which the initiative is delivered as intended in real-world settings rather than clinically controlled research settings	Examine intervention reliability and feasibility	Delivery of education and information sessions to staff (medical, nursing, AH) & interdisciplinary forums Provision of rehabilitation information brochures to patients on admission to the ward Provision of Rehab-Toolkit on the ward for easy accessibility and embedding assessment forms into medical records	Variable consistency of ‘Rehab-Toolkit’ use. Staff fidelity was gauged from the contents of assessments recorded in medical records Ward staff changes – need for continuous reminder system for rehabilitation referral and follow-up systems	Positive short-term impacts (admission to discharge) of Rehab-Toolkit on outcomes measured, demonstrated feasibility of the programme in real-world clinical practice Further larger and follow- up studies are required to demonstrate the true effect of these in the longer term The contents of the assessment tool can be reviewed regularly to remain relevant
Maintenance	Long-term effects of intervention on individual and settings	Plan maintenance and dissemination of the rehabilitation toolkit	No additional resources were allocated as the study involved rehabilitation staff who assessed patients as per routine care	Incorporation of functional assessment of patients into routine practice undertaken by staff	Widespread implementation of Rehab-Toolkit is beyond the scope of study and limited to some inpatient wards only Future broader programme is planned including the inclusion of the Rehab-Toolkit in EMR. Cost analysis was not within the scope of this study' cost-effectiveness analysis would be important if widespread imple-mentation of Rehab-Toolkit was to be considered

Adapted from: Song et al. (25).

## DISCUSSION

Improved cancer care has significantly increased patient survival rates; however, cancer-related (both condition and treatment-specific) morbidities are on the rise. There is global consensus amongst healthcare experts for the integration of rehabilitation into the cancer-care continuum due to high rates of cancer-related functional morbidity and relatively low utilization of rehabilitation services in cancer care settings (26–28). One cross‑sectional survey of rehabilitation health professionals in hospital and ambulatory care settings in Australia and New Zealand reported low uptake of cancer rehabilitation (29); only 17.7% worked in a dedicated cancer rehabilitation programme (29). To our knowledge, this is the first study to use the RE-AIM implementation framework as a process evaluation tool in cancer settings to identify barriers and implement strategies with contextual adaptations that can be easily adopted in routine clinical practice.

This quality improvement initiative addressed current challenges in the healthcare delivery process in the post-pandemic health system. Overall, the implementation of a structured rehabilitation assessment and referral system improved clinical team engagement with the rehabilitation professionals, and improved access to timely rehabilitation referrals, intervention, and better patient outcomes including physical function and quality of care. The study identified staff and systemic barriers in rehabilitation service provision in cancer care, and provided the clinical teams with measures to identify functional changes over time and better integration of individualized rehabilitation interventions throughout the patient care continuum. System and clinic-level policy and process changes included: the establishment of new staffing models such as discharge care coordinator roles, standardized patient functional assessment and referral systems (to other hospitals, rehabilitation facilities, and community providers), and development of user-friendly clinical pathways, the commencement of a new Rehabilitation Medicine Clinic, enhanced knowledge, awareness of rehabilitation amongst patients and healthcare professionals, and engagement of interdisciplinary stakeholders.

The integration of the rehabilitation functional assessment tool provided objective and subjective measures for early identification of patient needs, timely evidence-based intervention, and evaluation of functional changes over time. All assessments were conducted as per usual clinical practice between rehabilitation physicians and other clinical services, with no additional burden to the patients/staff and no additional costs. Information collected from complex care interdisciplinary meetings was already a routine practice for reporting to the PMCC Patient Flow Executive Team. Their descriptive characterization and experiences provided insight on the existing rehabilitation programme, including gaps in care that drove this improvement initiative, and the implementation of the rehabilitation input.

Cancer patients often have a complex interplay of various disease-specific factors (physical, social, psychosocial, and cognitive) that require an integrated interdisciplinary approach (30, 31). Diagnosis of cancer itself can have distressing psychological implications for patients (and families), and treatment regimens can be associated with considerable adverse effects (32). In the disease trajectory, patients can have various adjustment issues, such as employment, education, perceptions of self-worth, self-image, marital stress, etc. Patients (and families and carers) struggle to cope with increased care needs, financial constraints, and limitations in participation (32). These can have a cumulative effect and can lead to a negative impact on treatments/recovery and overall QoL, with significant socioeconomic implications due to increased demand for healthcare, social and vocational services, and caregiver burden (9, 10). Therefore, patients require adequate and timely assessment of their needs for evidence-based management in real-time, including rehabilitation.

Implementation science offers methodological approaches for investigating the landscape of clinical-care delivery issues, devising and carrying out plans to implement evidence-based interventions, and ensuring the continuity of those interventions over time (28, 33). Implementing change in clinical practice is challenging, and requires critical analysis of clinical context to equip clinicians with suitable decision support and a clinical information system (20, 28). This approach is consistent with the literature indicating that innovations most readily adopted are those compatible with current systems and that are user-friendly, align with the values, norms, and perceived needs of those adopting them, and empower users to experiment with them, adapting, refining, or modifying them to fit their requirements (33–35). The guiding principles underlying efforts to stimulate sustained cultural change in clinical settings include: aligning vision and action; making incremental changes within a comprehensive transformation strategy; fostering distributed leadership; promoting staff engagement; creating collaborative relationships; and continuously assessing and learning from change (35). In line with these, we provided structured assessment and care processes that aided treating clinicians in decision-making based on evidence and reporting systems that are user-friendly and accessible system-wide. This led to the early identification of impairments, identifying barriers to discharge from the hospital, and facilitating timely referrals to appropriate subacute services. This innovative model was feasible and fitted well into existing clinical care in a planned and strategic manner. Further, this project was tailored to the priorities and vision of the Learning Health System programme, i.e., using routinely collected clinical data to generate new knowledge (D2K), using knowledge to inform clinical practice (K2P), and evaluating changes in clinical practice (P2D) (36, 37).

### Limitations

There are several limitations in completing the study. This is a descriptive analytical study, without any comparator group, with a small selective cancer cohort from a single tertiary metropolitan centre. Therefore, contextual issues and differences in patient populations may limit the generalizability and validity of the findings to other centres. There are no patient outcome data available for pre–post implementation comparisons. We were not able to address the full extent of the RE-AIM framework, specifically, its “Maintenance” domain, as we did not evaluate the outcomes in the longer term. Further, we were not able to do a cost analysis of the programme. This was beyond the scope of the study. However, this pilot study provides preliminary data to support a future, large-scale trial to assess longer term patient outcomes. We also acknowledge that the presence of researchers in the staff meeting may influence discussions. However, the researchers are already part of the treating clinical team and did not adversely influence any decision-making, and the team as a whole made all decisions. No negative events arising from these activities were recorded.

### Conclusion

This pragmatic quality improvement study established the importance of integration of rehabilitation services in the cancer care continuum for proactive management of functional disability. It highlights the need to address the clinical and system-level barriers for effective implementation approaches that are feasible, and to identify practice-based improvement strategies to enhance the uptake and delivery of the required rehabilitation intervention. It is envisaged that findings can be leveraged to guide the implementation of rehabilitation-inclusive cancer care delivery. Future research is required to confirm the findings through robust and larger multi-centre studies to evaluate longer-term patient outcomes and alignment of treatment with patient needs.
